# Resveratrol-Loaded Polymeric Nanoparticles: Validation of an HPLC-PDA Method to Determine the Drug Entrapment and Evaluation of Its Antioxidant Activity

**DOI:** 10.1155/2013/506083

**Published:** 2013-10-24

**Authors:** Gabriela da Rocha Lindner, Najeh Maissar Khalil, Rubiana Mara Mainardes

**Affiliations:** Department of Pharmacy, Laboratory of Pharmaceutical Nanotechnology, Universidade Estadual do Centro-Oeste/UNICENTRO, Rua Simeão Camargo Varela de Sá 03, 85040-080 Guarapuava, PR, Brazil

## Abstract

Poly(lactic acid) (PLA) and PLA-poly(ethylene glycol) (PLA-PEG) nanoparticles containing resveratrol (RVT) were developed, and their antioxidant activity was evaluated. An analytical method using high performance liquid chromatography (HPLC)/photodiode array (PDA) detection was also developed and validated for RVT determination in nanoparticles. The mobile phase consisted of methanol : water (51 : 49, v/v) flowed at 0.9 mL/min, and the PDA detector was set at wavelength of 306 nm. The mean diameter of the nanoparticles varied between 180 and 220 nm, and the encapsulation efficiency of RVT ranged from 60% to 88%. The nanoparticles containing RVT were evaluated for their ability to scavenge the radical (2,2-azinobis (3-ethylbenzothiazoline-6-sulfonic acid) diammonium salt) (ABTS^•+^). The profile obtained from the PLA nanoparticles containing RVT demonstrated that after 24 h, there was almost no increase in antioxidant activity, which was lower than that of the free RVT and RVT-loaded PLA-PEG nanoparticles. For PLA-PEG nanoparticles, the radical-scavenging activity of RVT was shown to increase with time, and after 48 h, it was similar to that observed with free RVT.

## 1. Introduction

 The human body constantly produces reactive oxygen species that are generated as by-products of biological reactions or by exogenous factors derived from the metabolism of oxygen [1]. However, this production is balanced by endogenous antioxidants and enzymes, such as superoxide dismutase, catalase and glutathione peroxidase [2]. In extreme concentrations, cellular oxidative stress can induce DNA damage, leading to cancer, degenerative, and vascular diseases and others [3].

The use of exogenous antioxidant compounds to compensate for this imbalance has received great attention, mainly in natural product-based compounds. Among these, transresveratrol (3,4′,5-trihydroxystilbene, RVT), a polyphenolic compound found mainly in grapes, peanuts, and herbs, is rich in pharmacological activities. Studies demonstrate high antioxidant activity [4, 5], cardiovascular protective effects [6], antiviral activity [7], neuroprotective capacity (studied in metabolic disorders and neurodegenerative diseases such as Alzheimer's, Huntington's, and Parkinson's) [8], estrogenic functions [9], and potent antitumor activity [10] for this compound.

 Although there is therapeutic potential for this molecule, RVT presents pharmacokinetic drawbacks; for example, it is extensively metabolized after oral administration, resulting in low oral bioavailability. Additionally, a large portion of the dose is converted to conjugate sulfates, which is the limiting step in the systemic bioavailability of RVT [11]. The low aqueous solubility (log⁡⁡*P* of approximately 3.1) also favors the reduction of drug bioavailability, transforming its therapeutic and prophylactic potentials in a challenge.

 The use of colloidal drug carriers as polymeric nanoparticles is a strategy to combat these disadvantages. The physicochemical characteristics of nanoparticles influence the pharmacokinetics of the drug, affecting its bioavailability and biodistribution. Additionally, it promotes controlled and prolonged drug release to help reduce toxicity [12].

 Obtaining nanoparticles requires extensive characterization and determination of the drug content within the nanoparticles. This parameter must be properly verified because the drug must be efficiently loaded into the nanoparticles to reach its therapeutic goal. Therefore, a suitable and validated quantification method is required. Several analytical methods have been developed to quantify RVT in samples, such as plasma, urine, wine, and butter; however, few analytical methods have been reported for the determination of RVT in nanoparticles. UV-Vis spectroscopy [13–15] and HPLC-UV/Vis methods [16–20] have been reported for such determination, but these chromatographic methods have not been validated and only address the mobile phase and other basic parameters. In this work, a reverse-phase HPLC-PDA method was developed and validated for the rapid, simple, and optimized determination of the encapsulation efficiency of RVT in poly(lactic acid) (PLA) and PLA blends with poly(ethylene glycol) (PLA-PEG) nanoparticles. Additionally, the nanoparticles containing RVT were evaluated for their ability to scavenge the radical (2,2-azinobis (3-ethylbenzothiazoline-6-sulfonic acid) diammonium salt) (ABTS^●+^).

## 2. Materials and Methods

### 2.1. Materials

 Trans-RVT was obtained from Pharmanostra (Brazil). PEG (10 kDa), PLA (85,000–160,000 Da), and polyvinyl alcohol (PVA, 31 KDa, and 88% hydrolyzed) were purchased from Sigma-Aldrich (USA). Ethyl acetate (P.A) and dimethyl sulfoxide (P.A, DMSO) were purchased from Biotec (Brazil), and dichloromethane was purchased from FMaia (Brazil). HPLC-grade methanol was purchased from J.T. Baker (USA). Water was purified using a Milli-Q Plus system (Millipore) with a conductivity of 18 MΩ. ABTS (2,2-azinobis (3-ethylbenzothiazoline-6-sulfonic acid) diammonium salt) and potassium persulfate (dipotassium peroxydisulfate) both were obtained from Sigma-Aldrich (USA).

### 2.2. Equipment

 The HPLC system consisted of a Waters 2695 Alliance (Milford, MA, USA) combined with a photodiode array wavelength detector (PDA) (Waters 2998). This system was equipped with a quaternary pump, an autosampler, an online degasser, and a column compartment with temperature control. Data acquisition, analysis, and reporting were performed using the Empower chromatography software (Milford, MA, USA). The analysis was conducted using a reverse phase C18 column (Xterra Waters) with a 5 *μ*m particle size, 4.6 mm internal diameter, and 250 mm length. 

### 2.3. Chromatographic Conditions

Chromatographic analyses were performed in the isocratic mode with a mobile phase consisting of a methanol and water mixture (51 : 49, v/v) pumped at a flow rate of 0.9 mL/min. The sample injection volume was 20 *μ*L, and the PDA was set at 306 nm. The method run time was 6.4 min at a temperature of 25°C.

### 2.4. Preparation of Standard and Sample Solutions

 An RVT stock standard of 1 mg/mL was prepared in a methanol : water mixture (50 : 50, v/v), and subsequent dilutions were carried out to obtain six standard solutions (10, 20, 25, 30, 40, and 50 *μ*g/mL). Additionally, six standard solutions (1, 2, 4, 6, 8, and 10 *μ*g/mL) were obtained to determine the limit of quantification (LOQ) and the limit of detection (LOD). 

 Prior to injection, the standards and samples were filtered through a 0.22 *μ*m pore-size filter (Millipore, Bedford, USA).

### 2.5. Method Validation

 The HPLC method was validated in terms of specificity, linearity, precision (intra- and interday), accuracy, robustness, LOD, and LOQ according to the International Conference on Harmonization (ICH) guidelines [21].

 The specificity was evaluated by comparing the representative chromatograms of samples containing possible interfering substances (excipients used in nanoparticle composition) and samples containing RVT. Additionally, specificity was demonstrated by performing stress studies (i.e., light stability, pH, and temperature variation).

 The linearity was determined by calculating a regression line from the plot of the peak area versus concentration for the six standard solutions in a 50 : 50 (v/v) methanol : water mixture (10, 20, 25, 30, 40, and 50 *μ*g/mL) using a linear least-squares regression.

 Precision was assessed at two levels: repeatability and interday variability. The repeatability of the measurements was assessed by testing three different standard solutions (10, 25 and 50 *μ*g/mL, *n* = 10) during the same day. The interday precision was evaluated by analyzing three different standard samples (10, 25, and 50 *μ*g/mL, *n* = 3) on three different days. The results were reported as the standard deviation (SD) and relative standard deviation (RSD). 

 The accuracy was determined by calculating the percent recovery of the RVT at three concentration levels and then determining the RSD. The mean concentration value obtained for each level was compared to the theoretical value, which was considered to be 100%.

 The robustness was evaluated by deliberately varying the temperature of the analytical column (20 or 30°C), while using a standard C18 column (5 *μ*m particle size, 4.6 mm internal diameter, and 250 mm length from Vertical Chromatography Co.).

 The LOD and LOQ were determined from the specific calibration curve obtained using six standard solutions (1, 2, 4, 6, 8, and 10 *μ*g/mL). The following equations (1) were used according to ICH [21]:

(1) $$\begin{array}{c} LD=3.3\times \frac{\sigma }{S},\\ LQ=10\times \frac{\sigma }{S}, \end{array}$$

where *σ* is the standard deviation of the response and *S* is the slope of the calibration curve.

### 2.6. Method Applicability

#### 2.6.1. Determination of RVT Encapsulation Efficiency in PLA and PLA-PEG Blended Nanoparticles

The RVT-loaded nanoparticles were obtained by a single-emulsion solvent evaporation technique and subsequently lyophilized and stored in a light-protecting container. PLA was dissolved in dichloromethane either with or without PEG at room temperature, and the RVT were then added to the solution. This solution was poured rapidly into a PVA aqueous solution (1%, w/v) and emulsified by means of sonication for 10 min (80–100% of 500 W, Unique Ultrasonic Mixing, mod. DES 500, equipped with a 4 mm probe, Unique Group, Brazil), which resulted in an oil-in-water (O/A) emulsion. Next, the organic solvent was rapidly removed by evaporation under vacuum at 37°C (20 min). The particles were then recovered by ultracentrifugation (19,975 g, 30 min, 4°C, Cientec CT-15000R centrifuge, Brazil) and washed twice with water to remove the surfactant. The nanoparticles were dispersed in the cryoprotectant sucrose (5%, w/v), and the resulting nanosuspension was subsequently cooled to −18°C and freeze-dried (Terroni, Brazil).

The mean particle size, size distribution, and polydispersity index were determined by dynamic light scattering (BIC 90 plus, Brookhaven Instruments Corp.). The analyses were performed at a scattering angle of 90° and a temperature of 25°C. For each sample, the mean particle diameter, polydispersity, and standard deviation for ten determinations were calculated.

The amount of RVT in the nanoparticles was determined indirectly. The supernatant obtained from the ultracentrifugation process was diluted in the mobile phase (1 : 1000), filtered through a 0.22 *μ*m pore-size filter and analyzed by the HPLC method previously developed and validated. The drug concentration in the supernatant was obtained by comparing the concentration to a previously constructed analytical curve. The amount of RVT trapped in the nanoparticles was determined by subtracting the quantity in the supernatant from the total quantity used during the preparation. These analyses were performed in triplicate.

### 2.7. Nanoparticles Applicability 

#### 2.7.1. Antioxidant Activity Assay

The cation radical ABTS^●+^ was employed to measure the antioxidant activity of free RVT, RVT-loaded nanoparticles (PLA and PLA-PEG nanoparticles), and blank nanoparticles.

A mixture of ABTS (7 mM) and potassium persulfate (2.45 mM) was prepared and allowed to stand at room temperature [22, 23]. The ABTS^●+^ solution was diluted to an absorbance of 0.70 at 734 nm in a 50 mM phosphate buffer, pH 7.4. Blank nanoparticles and different concentrations of RVT (free or nanoencapsulated) ranging from 1 to 25 *μ*M were used. The compounds were incubated at 37°C under constant agitation and protected from light for 0, 24, 48, and 72 h. After incubation at 37°C, aliquots with known concentrations were incubated for 30 min with ABTS^●+^, and the absorbance was measured at 734 nm. The control used during this assay was the buffer solution. The percentage of inhibition was calculated by

(2) $$\begin{array}{c} \%\text{Inhibition}=\frac{\left(Ac\times At\right)}{Ac}\times 100, \end{array}$$

where *Ac* is the absorbance of control and *At* is the absorbance of test.

The concentration of RVT that resulted in 50% of inhibition of ABTS^●+^ (IC_50_) was calculated by the linear regression of the RVT concentration versus percentage of ABTS^●+^ inhibition curves.

## 3. Results

### 3.1. Method Development

**Figure 1:**
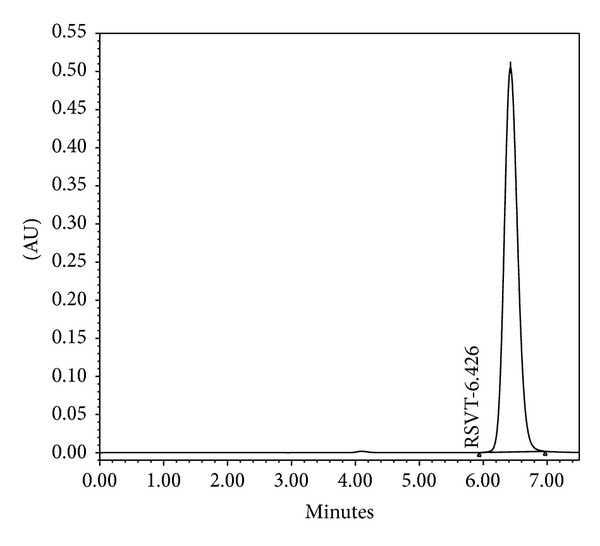
Representative HPLC chromatograms of RVT standard (40 *μ*g/mL) in methanol : water. Mobile phase, methanol : water (51 : 49, v/v); flow rate, 0.9 mL/min; PDA detection wavelength, 306 nm; column temperature, 25°C; injection volume, 20 *μ*L.

Initial runs were performed using a mobile phase mixture of methanol and acetonitrile based on existing methods for RVT quantification in plasma [24]. Various ratios in the isocratic mode were tested, some of which led to the presence of more than one peak (methanol : acetonitrile 3 : 1 and 1.5 : 1). Consequently, water and acetic acid were introduced as a new mobile phase mixture [25, 26]. A few symmetric peaks were achieved with methanol, acetonitrile, water, and acetic acid; however, neither method demonstrated repeatability or accuracy. When acetonitrile and acetic acid were removed and only methanol and water were used, lower tailing and more symmetrical peaks were observed. The best peak with respect to width and symmetry was observed with a mobile phase of water and methanol in the ratio of 49 : 51 (v/v) and a flow rate of 0.9 mL/min. The peak was detected at 6.4 min (Figure 1).

### 3.2. Method Validation

#### 3.2.1. Linearity

Linearity was evaluated at six concentration levels (10–50 *μ*g/mL) by calculating the following regression equation and the correlation coefficient (*r*) using the least-squares method:

(3) $$\begin{array}{c} Y=1.54\times 10^{5}A+5.36\times 10^{4},\\ r=0.9999, \end{array}$$

where *Y* is the peak area and *A* is the standard solution concentration in *μ*g/mL. An *r*-value near 1 indicates linearity in the proposed range.

 The validity of the assay was confirmed by an analysis of variance, which showed that the linear regression was significant and that the deviation from linearity was not significant (*P* < 0.01).

#### 3.2.2. Accuracy

Accuracy was assessed by calculating the percent recovery and the RSD of the mean concentration of the analyte at three different concentrations. Three standard solutions (10, 30, and 50 *μ*g/mL) were carefully prepared in triplicate and analyzed using the previously proposed method. Detailed results are presented in Table 1. The mean percent recovery of RVT from the samples was 99.30% (RSD = 2.43%, *n* = 9). The results show agreement between the experimental and theoretical values.

#### 3.2.3. Precision

The precision is a measure of the relative errors of the method, expressed as the RSD for repeatability and intermediate precision. Three concentrations of RVT (10, 30, and 50 *μ*g/mL) were prepared in triplicate and analyzed on one or three different days to evaluate intraday or interday variation, respectively. The RSDs of the responses were calculated for each case and are shown in Table 2. The results indicate that precision was achieved because the maximum RSD value obtained was 1.51%.

#### 3.2.4. Robustness

Robustness is a measure of the influence of small changes to the analytical procedures/parameters on the response. The robustness was evaluated based on the percent recovery and RSD values obtained using different parameters for column temperature and commercial mark (Table 3). The method was robust with regard to these alterations in the chromatographic parameters. The maximum RSD obtained was 2.44%.

#### 3.2.5. Limit of Quantification and Limit of Detection

In the present study, the lowest concentration at which an analyte can be detected (LOD) or quantified (LOQ) with acceptable precision and accuracy was calculated from the SD of the response and the slope obtained from linear regression of a specific calibration curve (1–10 *μ*g/mL) in the low-end region of the proposed range [21]. The method was found to be linear in this range with an *r*-value of 0.999. The LOD and LOQ were found to be 68.0 and 229.0 ng/mL, respectively.

#### 3.2.6. Specificity

**Figure 2:**
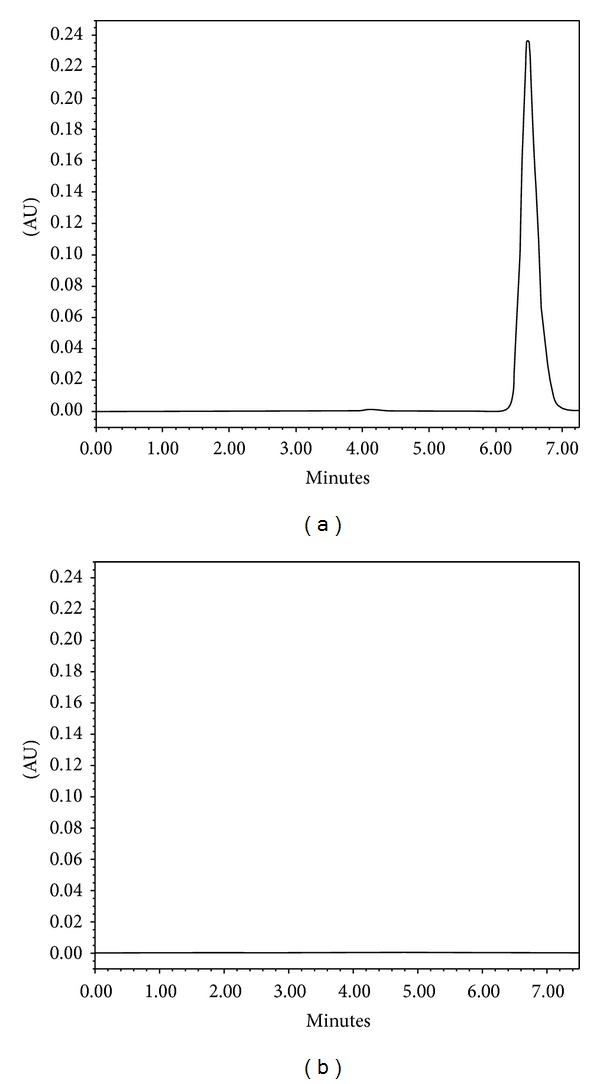
Representative HPLC chromatograms of RVT sample in supernatant from nanoparticles (a) and supernatant from blank nanoparticles (b). Conditions: mobile phase, methanol : water (51 : 49, v/v); flow rate, 0.9 mL/min; PDA detection wavelength, 306 nm; column temperature, 25°C; injection volume, 20 *μ*L.

The specificity of the method was evaluated by comparing the chromatograms of RVT standards and samples to those with potential interfering formulation components. For this purpose, blank nanoparticles (drug-unloaded nanoparticles) were prepared as described in Section 2.6.1, and the supernatant obtained after centrifugation was diluted in a methanol : water mixture (50 : 50, v/v) and analyzed by the described HPLC method. The representative chromatogram of the RVT sample (Figure 2(a)) showed the RVT peak at approximately 6.4 min, which was in agreement with that obtained for the RVT standard (Figure 1). No peaks at this retention time were observed in the chromatogram of the supernatant from the blank nanoparticles (Figure 2(b)), which indicates that there was no interference in the quantitative determination of RVT from the formulation components. 

Tests were also performed under stress conditions (i.e., temperature, visible light, and pH variation) to detect the occurrence of possible interfering peaks at 306 nm resulting from the degradation of RVT. The results showed no alterations in the RVT retention time when the sample was exposed to temperature, visible light, and acid medium. However, a decrease in RVT recovery caused by photodegradation was observed. Exposure to the alkaline conditions resulted in sample degradation, making RVT peak identification impossible. Additionally, in all stress conditions evaluated, no peaks for RVT metabolites were observed. This method can be considered highly specific because no potential interfering peak was observed.

### 3.3. Method Applicability

The proposed method was applied to the analysis of RVT in PLA and PLA-PEG nanoparticles and serves as a tool for the determination of the encapsulation efficiency without any interference, as demonstrated in the specificity assay.

The single-emulsion solvent evaporation method was successfully developed for obtaining PLA and PLA-PEG nanoparticles containing RVT. The mean diameter of the PLA and PLA-PEG nanoparticles was approximately 227.56 ± 9.57 nm and 185.46 ± 1.65 nm, respectively, with both exhibiting a monomodal distribution profile. The encapsulation efficiency of RVT was 82.47 ± 5.8% (*n* = 3) in the PLA nanoparticles and 68.54 ± 9.08% (*n* = 3) in the PLA-PEG nanoparticles. Statistical treatment by *t*-test (*P* < 0.05), showed no significance in the encapsulation efficiency.

### 3.4. Nanoparticles Applicability

The nanoparticles containing RVT were evaluated for their ability to scavenge the radical ABTS^●+^. The results of radical inhibition percentage obtained from the 1, 5, 10, 20, and 25 *μ*M RVT concentrations, free or encapsulated in two different nanoparticles formulations, were evaluated at times 0, 24, 48, and 72 h and are presented in Table 4.

At 0 h, it can be observed that the two nanoparticle formulations exhibit the same ability to scavenge ABTS^●+^ at all the concentrations tested (*P* > 0.05). The free RVT presented a higher percentage of ABTS^●+^ inhibition compared to the nanoparticles (*P* < 0.05). At 24 h, the two nanoparticles formulations showed the same profile presented for previous time, with the exception of the RVT-loaded PLA-PEG nanoparticles (25 *μ*M), which showed an increase in ABTS scavenging (*P* < 0.05). The free RVT, in the three lower concentrations (1, 5, and 10 *μ*M), decreased the ABTS^●+^ scavenging ability (*P* < 0.05); however, at 20 and 25 *μ*M, the percentage of radical inhibition was similar to the previous time (*P* > 0.05). At 48 h, there was a significant increase in the radical inhibition by PLA-PEG nanoparticles, while for the PLA nanoparticles the profile was the same as that observed at 24 h. At 25 *μ*M, the two nanoparticles present similar responses (*P* > 0.05), but only RVT-loaded PLA-PEG nanoparticles were so effective as free RVT (*P* > 0.05). At 72 h, in higher RVT concentration, it can be observed that the RVT-loaded PLA-PEG nanoparticles presented the same scavenger activity as free RVT (*P* > 0.05), but the response obtained with the RVT-loaded PLA nanoparticles was inferior (*P* < 0.05). In general, we can affirm that the two higher RVT concentrations (20 and 25 *μ*M) resulted in better scavenger activity for the free RVT and RVT-loaded nanoparticles. The profile obtained with the PLA nanoparticles containing RVT demonstrated that after 24 h, there was almost no increase in response. However, the profile obtained with the PLA-PEG nanoparticles demonstrated that the RVT response increased with time.

The IC_50_ of RVT scavenging ABTS^●+^ as a function of time was obtained, and the results are shown in Table 5. The free RVT showed an increase in IC_50_ with time. The values of IC_50_ obtained with the RVT-loaded PLA nanoparticles presented few variations with time and were superior to those obtained with free RVT (*P* < 0.05). The values of IC_50_ obtained with the RVT-loaded PLA-PEG nanoparticles were decreased with time and, after 48 and 72 h, were considered similar to free RVT (*P* > 0.05).

## 4. Discussion

The polyphenol RVT is extensively used for pharmaceutical applications and has received great attention in recent years due to its prophylactic and therapeutic abilities against reactive oxygen species. However, its low aqueous solubility and high metabolism significantly decrease its bioavailability. Polymeric nanoparticles have been proven to increase the therapeutic benefits of drugs, decrease the toxic effects of drugs, and deliver the drug to a specific site of action. The physicochemical parameters of nanoparticles influence the pharmacokinetics of the drug, affecting its bioavailability and biodistribution [12, 27]. Based on this, RVT could be used as a drug for minimizing or preventing oxidative stress, and its carrier by the polymeric nanoparticles could generate many pharmacokinetic benefits to the drug without loss of the biological potential of this molecule.

The main objective of this study was to develop an analytical method coupled with a PDA detector to quantify RVT loaded in PLA and PLA-PEG nanoparticles by the indirect method. This quantification method is extensively used [12, 28, 29] for its speed and ease, compared to direct methods (dissolution of a polymeric matrix and drug extraction) because it allows the analysis of this factor even before the freeze-dried process.

Several analytical methods are described in the literature with the purpose of quantifying RVT in samples, such as wines [26, 30, 31], plasma [24], urine [32], tissues [33], and peanuts [34]. 

The literature mainly describes spectrophotometric methods for RVT quantification in nanoformulations [13–15]; however, these methods are not as sensitive as the HPLC methods. The few studies using HPLC-UV/Vis, proposed by Shao et al. [17] and Lu et al. [16] and collaborators, use a mobile phase mixture of methanol, double-distilled water, and glacial acetic acid (48/52/0.05, v/v/v) to quantify RVT in biodegradable nanoparticles. Gokce et al. [19] used a very similar mobile phase, composed of methanol, water, and acetic acid (52 : 48 : 0.05 v/v/v). Sanna et al. [18] quantified RVT in nanoparticles with a very complex mobile phase: A : B (21 : 79, v/v), where solvent A was trifluoroacetic acid (TFA) in water (0.1/99.9 v/v) and solvent B was acetonitrile/TFA/water (95/0.07/4.93 v/v). The analyte was eluted at a flow rate of 0.2 mL/minute in an isocratic elution period of 25 min. Lee et al. [20] described a mobile phase composed of 25 mM potassium dihydrogen phosphate buffer and acetonitrile (50 : 50, v/v) for RVT quantification in nanoparticles. The cited methods only cite the mobile phase and other basic parameters used, but they do not detail the method validation or give any information about peak characteristics or retention times.

The HPLC method developed and validated in this work represents an alternative to these other methodologies and satisfies the requirement for detailed data in the literature for analyzing RVT in nanoparticles via HPLC-PDA detection. The short retention time of RVT allows for the analysis of a large number of samples in a short period of time and reduces costs because of the solvents used and the absence of buffer in the composition of the mobile phase.

The proposed method was applied to the analysis of RVT in PLA and PLA-PEG nanoparticles. The nanoparticles were successfully obtained by the single-emulsion solvent evaporation method, which is ideal for a hydrophobic drug such as RVT. The particles presented nanometric sizes and high encapsulation efficiency. The antioxidant activity of RVT in nanoparticles was evaluated by the ABTS^●+^ assay. In the analysis of the IC_50_ values, we observed that better results for RVT-loaded PLA-PEG nanoparticles were obtained with time, probably due to the prolonged drug release characteristics promoted by the nanoparticles, but this profile was not observed with PLA nanoparticles, since the IC_50_ at 0 h was the same as that in 72 h. At 48 and 72 h, the free RVT and RVT-loaded PLA-PEG nanoparticles presented the same efficacy. Comparing the two nanoparticles, the difference between the results obtained by PLA and PLA-PEG formulations can be explained by the presence of PEG. PEG is able to modify the amount of drug released from the polymeric matrix. In recent work, we observed that the drug release profile from PLA-PEG nanoparticles was faster than that observed from PLA nanoparticles. This difference can be attributed to the enhancement of water permeation and drug diffusion through the polymeric matrix because of the hydrophilicity of PEG. The presence of PEG causes the polymeric matrix to become more amphiphilic than the PLA matrix, while the wettability of the nanoparticle surface also increases. These characteristics contribute to an increase in the drug release profile [29].

These results indicate that PLA and PLA-PEG nanoparticles are potential carriers for RVT. Despite the fact that RVT-loaded PLA nanoparticles demonstrated inferior antioxidant ability compared to PLA-PEG nanoparticles and free RVT, and the RVT from PLA-PEG nanoparticles exhibited the same antioxidant activity as free RVT only after 48 h; we must consider the advantages of drug-loaded nanoparticles over free drug, such as improved pharmacokinetics, prolonged and controlled drug release. The *in vivo* performance of the drug-loaded nanoparticles is superior to that of a free drug. The nanoencapsulation of RVT could improve its pharmacokinetics and solubility; therefore, the potential pharmacological properties of this molecule should be further explored.

## 5. Conclusion

The reverse-phase HPLC method using PDA detection was developed and validated according to the guidelines of ICH and was shown to be fast, simple, and reliable during the determination of the encapsulation efficiency of RVT in PLA and PLA-PEG nanoparticles. The RVT-loaded nanoparticles, especially in PLA-PEG nanoparticles, were very effective as a scavenger of the ABTS radical, suggesting that the polymeric nanoparticles could be used as RVT carriers for applications in prophylaxis or the treatment of diseases involving oxidative stress. More studies are necessary to test this hypothesis.

## Figures and Tables

**Table 1:** Accuracy results for the RVT concentrations in standard solutions (*n* = 3).

RVT standard solution (*μ*g/mL)	Recovery (%)	RSD (%)
10	96.49	0.09
30	100.69	1.25
50	100.73	0.09

RSD: relative standard deviation.

**Table 2:** Precision results for the different levels of RVT in standard solutions.

RVT standard solution (*µ*g/mL)	Measured concentration ± SD^a^ (*µ*g/mL)	RSD^b^ (%)
Analysis repeatability (*n* = 10)		
10	10.08 ± 0.03	0.32
30	30.60 ± 0.05	0.16
50	49.80 ± 0.06	0.12

Intermediate precision (*n* = 3)		
Day 1		
10	9.65 ± 0.04	0.43
30	30.21 ± 0.38	1.25
50	50.36 ± 0.04	0.09
Day 2		
10	10.03 ± 0.02	0.17
30	30.62 ± 0.11	0.35
50	49.87 ± 0.14	0.29
Day 3		
10	9.53 ± 0.14	1.51
30	29.82 ± 0.11	0.37
50	50.15 ± 0.09	0.18

^a^SD: standard deviation; ^b^RSD: relative standard deviation.

**Table 3:** Percentage of recovery and RSD obtained in the analysis of robustness after changes in original temperature of the method (25°C) and column mark (*n* = 3).

Changes to original method	Percentage of recovery ± RSD^a^			
	10 *µ*g/mL	30 *µ*g/mL	50 *µ*g/mL	Mean
Temperature 30°C	101.05 ± 0.74	101.43 ± 1.00	101.32 ± 0.93	101.27 ± 0.15
Temperature 20°C	99.36 ± 0.45	100.58 ± 0.41	99.64 ± 0.25	99.86 ± 0.10
Similar column	102.86 ± 1.99	103.51 ± 2.44	102.46 ± 1.72	102.94 ± 0.53

^a^RSD: relative standard deviation.

**Table 4:** Percentage of inhibition of the radical ABTS^●+^ from free and nanoencapsulated RVT in concentrations of 1, 5, 10, 20, and 25 *µ*M for 0, 24, 48, and 72 h.

RVT concentration (*µ*M)	Inhibition of ABTS^●+^ (%)		
	RVT-loaded PLA nanoparticles	RVT-loaded PLA-PEG nanoparticles	Free RVT
0 h			
1	6.84 (±1.04)^a^	8.95 (±1.70)^a^	13.29 (±1.25)^b^
5	28.56 (±9.27)^a,b^	15.38 (±0.76)^a^	46.33 (±2.66)^b^
10	35.50 (±1.58)^a^	33.23 (±5.18)^a^	69.21 (±1.58)^b^
20	53.35 (±3.88)^a^	56.40 (±2.63)^a^	95.48 (±4.61)^b^
25	65.54 (±7.30)^a^	64.00 (±5.42)^a^	99.70 (±0.29)^b^

24 h			
1	5.67 (±0.45)^a^	2.92 (±2.79)^a^	7.35 (±2.27)^a^
5	10.89 (±0.58)^a^	14.97 (±3.87)^a^	28.68 (±3.29)^b^
10	22.75 (±1.87)^a^	24.09 (±1.57)^a^	49.35 (±5.10)^b^
20	50.49 (±4.17)^a^	55.66 (±1.65)^a^	95.46 (±5.20)^b^
25	65.75 (±3.14)^a^	75.87 (±5.86)^b^	97.88 (±1.91)^c^

48 h			
1	2.90 (±2.33)^a,c^	7.50 (±0.95)^b^	2.21 (±1.98)^c^
5	7.87 (±1.07)^a^	21.76 (±2.17)^a^	25.47 (±12.53)^a^
10	20.82 (±2.30)^a^	35.01 (±4.68)^b^	47.74 (±4.56)^c^
20	45.76 (±5.92)^a^	62.14 (±320)^b^	88.40 (±0.88)^c^
25	69.07 (±12.22)^a^	85.13 (±2.52)^a,b^	98.34 (±0.68)^b^

72 h			
1	12.13 (±0.65)^a^	14.01 (±1.07)^a^	3.83 (±0.76)^b^
5	13.30 (±2.19)^a^	25.22 (±1.67)^b^	28.30 (±1.96)^b^
10	24.90 (±4.45)^a^	40.58 (±4.30)^b^	51.43 (±3.25)^c^
20	57.26 (±13.78)^a^	70.45 (±2.39)^b^	92.17 (±4.86)^c^
25	76.43 (±2.49)^a^	90.99 (±3.45)^b^	95.52 (±1.58)^b^

^a,b,c^Mean of triplicate ± standard deviation analyzed per line. Same letters mean statistical equality, and inequality stats are indicated for different letters (ANOVA with Tukey post test and *α* 0,05).

**Table 5:** IC_50_ of RVT (free or nanoencapsulated) over the capture of the ABTS^●+^ cation radical in sodium phosphate buffer (50 mM, pH 7.4) and in the absence of light at room temperature (*ƛ* = 734 nm).

Time (h)	IC_50_		
	RVT-loaded PLA nanoparticles ± RSD*	RVT-loaded PLA-PEG nanoparticles ± RSD*	Free RVT ± RSD*
0	17.30 ± 0.71^a^	18.15 ± 0.95^a^	8.21 ± 0.41^b^
24	19.52 ± 1.08^a^	17.31 ± 0.70^b^	10.79 ± 028^c^
48	19.83 ± 2.48^a^	14.65 ± 0.10^b^	11.58 ± 0.78^b^
72	16.61 ± 1.08^a^	12.76 ± 0.80^b^	11.15 ± 0.34^b^

^a,b,c^Mean of triplicate ± standard deviation analyzed per line. Same letters mean statistical equality and inequality stats are indicated for different letters (ANOVA with Tukey post test and *α* 0,05).

*RSD: relative standard deviation.
